# Artificial Intelligence in Omics

**DOI:** 10.1016/j.gpb.2023.01.002

**Published:** 2023-01-12

**Authors:** Feng Gao, Kun Huang, Yi Xing

**Affiliations:** 1 Department of Physics, School of Science, Tianjin University, Tianjin 300072, China; 2 Frontiers Science Center for Synthetic Biology and Key Laboratory of Systems Bioengineering (Ministry of Education), Tianjin University, Tianjin 300072, China; 3 Department of Biostatistics and Health Data Science, Indiana University School of Medicine, Indianapolis, IN 46202, USA; 4 IUPUI Fairbanks School of Public Health, Indianapolis, IN 46202, USA; 5 Regenstrief Institute, Indianapolis, IN 46202, USA; 6 Center for Computational and Genomic Medicine, The Children’s Hospital of Philadelphia, Philadelphia, PA 19104, USA; 7 Department of Biomedical and Health Informatics, The Children’s Hospital of Philadelphia, Philadelphia, PA 19104, USA; 8 Department of Pathology and Laboratory Medicine, Perelman School of Medicine, University of Pennsylvania, Philadelphia, PA 19104, USA

Artificial intelligence (AI) is a powerful approach for solving complex problems in the processing, analysis, and interpretation of omics data, as well as the integration of multi-omics and clinical data. In recent years, AI has enabled remarkable breakthroughs across diverse biomedical fields, such as genomic variant interpretation, protein structure prediction, disease diagnosis, and drug discovery. Aiming to provide a forum for advances in the development and application of AI-based tools in omics, we have organized a special issue “Artificial Intelligence in Omics” for the journal *Genomics, Proteomics & Bioinformatics* (GPB). This special issue covers a broad spectrum of topics, including but not limited to: 1) AI-based models, methods, and software for the processing, analysis, visualization, and interpretation of omics data; 2) AI-based algorithms for the integrative analysis of omics, clinical, and health data, including biomedical images; 3) AI-based platforms for improving disease diagnosis, precision medicine, and patient care; and 4) AI-based approaches for protein structure prediction, gene function prediction, and drug discovery.

With enthusiastic responses to our call for submissions, we are pleased to announce that 15 articles have been selected for publication in this special issue, including four review articles, two original research articles, and nine method articles. A list of original studies and tools reported in this special issue is provided in Table 1.

**Table 1:** **Original studies and tools reported in the special issue on artificial intelligence in omics**

**Tool name**	**Short description**	**Link**	**Ref.**
–	Assessing and optimizing explainable ML models applied to transcriptomic data	https://github.com/zhaopage/model_interpretability	[5]
SOPHIE	Separating common and specific transcriptional responses using generative neural networks	https://github.com/greenelab/generic-expression-patterns https://github.com/greenelab/sophie	[6]
DGMP	Identifying cancer driver genes from multi-omics pan-cancer data	https://github.com/NWPU-903PR/DGMP https://ngdc.cncb.ac.cn/biocode/tools/BT007338	[7]
scEMAIL	Generating universal and source-free annotations for single-cell RNA-seq data with novel cell-type perception	https://github.com/aster-ww/scEMAIL https://ngdc.cncb.ac.cn/biocode/tools/BT007335	[8]
DeeReCT-TSS	Annotating TSSs in multiple cell types based on DNA sequence and RNA-seq data	https://github.com/JoshuaChou2018/DeeReCT-TSS_release https://ngdc.cncb.ac.cn/biocode/tools/BT007316	[9]
TIST	Analyzing transcriptome data and histopathological images integratively for spatial transcriptomics	http://lifeome.net/software/tist/ https://ngdc.cncb.ac.cn/biocode/tools/BT007317	[10]
DeepNoise	Disentangling signal and noise by DL-based classification of fluorescent microscopy images	https://github.com/Scu-sen/Recursion-Cellular-Image-Classification-Challenge https://ngdc.cncb.ac.cn/biocode/tools/BT007332	[11]
MAPD	Predicting protein degradability using ML analysis of protein-intrinsic features	https://github.com/liulab-dfci/MAPD https://mapd.cistrome.org	[12]
NetBCE	Predicting linear B-cell epitopes using an interpretable deep neural network	https://github.com/bsml320/NetBCE https://ngdc.cncb.ac.cn/biocode/tools/BT007321	[13]
TripletGO	Predicting gene functions by integrating transcript expression profiles with protein homology inferences	https://zhanggroup.org/TripletGO https://ngdc.cncb.ac.cn/biocode/tools/BT007277	[14]
DrSim	Enabling transcriptional phenotypic drug discovery by similarity learning	https://hub.docker.com/r/bm2lab/drsim/ https://github.com/bm2-lab/DrSim/ https://ngdc.cncb.ac.cn/biocode/tools/BT007273	[15]

*Note*: ML, machine learning; DL, deep learning; TSS, transcription start site.

Among the four review articles, Brendel et al. provided an overview of the application of deep learning (DL) models to single-cell RNA sequencing (scRNA-seq) data analysis, and discussed the current challenges and future opportunities in this field [1]. Stanojevic et al. presented an in-depth review on the computational methods for integrating multi-omics data from the same single cells or aligning multi-modal data from different cells, providing a detailed technical summary of currently available methods [2]. Li et al. surveyed machine learning (ML) approaches in lung cancer research, highlighting the challenges and opportunities for integrating complex biomedical data to improve lung cancer diagnosis and therapy [3]. Zha et al. reviewed current methods for microbiome data mining and knowledge discovery, with a focus on AI methods for elucidating microbial communities and their spatiotemporal dynamic patterns [4].

In an original research article, Zhao et al. studied the application of explainable ML models to transcriptomics data. They performed a comprehensive evaluation of multiple explainers and proposed optimization strategies to improve model reproducibility and interpretability. This work provides new insights and guidelines on the use of explainable ML models for exploring novel biological mechanisms [5].

Most of the articles in this special issue are method articles, reporting AI-based tools for various omics applications. Lee et al. introduced SOPHIE (Specific cOntext Pattern Highlighting In Expression data), which uses a generative neural network to separate common and context-specific transcriptional patterns. SOPHIE can distinguish common differentially expressed genes (DEGs) that are frequently altered across different biological contexts, and context-specific DEGs that are relevant for particular experimental conditions [6].

Zhang et al. reported DGMP (Directed Graph convolutional network and Multilayer Perceptron), a novel ML-based method for identifying cancer driver genes from multi-omics pan-cancer data. DGMP combines directed graph convolutional network to make use of diverse gene features and regulatory information in the multi-omics data, and multilayer perceptron to weigh preferentially on gene features. DGMP outperforms multiple state-of-the-art methods and identifies non-mutated cancer driver genes harboring epigenetic or expression alterations [7].

Wan et al. presented scEMAIL (Expert enseMble novel cell-type perception and local Affinity constraInts of muLti-order for scRNA-seq data). scEMAIL is a universal and source-free transfer learning-based annotation framework for scRNA-seq data. It can automatically identify novel cell types without using source data [8].

Zhou et al. reported DeeReCT-TSS (Deep Regulatory Code and Tools-Transcription Start Site), a DL-based method for genome-wide prediction of transcription start sites (TSSs). DeeReCT-TSS incorporates both DNA sequence data and conventional RNA-seq data as inputs, and substantially outperforms existing methods for TSS prediction based on DNA sequence data alone [9].

Shan et al. presented TIST (transcriptome and histopathological image integrative analysis for spatial transcriptomics), a novel analytical tool for spatial transcriptomics (ST) data. By integrating matched ST data and histopathological images, TIST identifies spatial clusters and enhances spatial gene expression patterns. TIST outperforms multiple start-of-the-art methods, as benchmarked on both simulated and real datasets [10].

Yang et al. developed DeepNoise, a semi-supervised DL-based model to distinguish true biological signals from experimental noise. The authors used DeepNoise to identify and classify the phenotypic effect of 1108 genetic perturbations based on 125,510 fluorescent microscopy images, achieving a high performance among competing methods [11].

In another original research article, Zhang et al. developed MAPD (model-free analysis of protein degradability), a ML method to predict protein degradability via protein-intrinsic features. MAPD achieves a high accuracy in predicting kinases that may be subject to targeted protein degradation, and may generalize to non-kinase proteins. The authors also identified important features predictive of protein degradability [12].

Xu and Zhao curated a large benchmark dataset of linear B-cell epitopes (BCEs), which play a critical role in immune responses. Based on this dataset, the authors developed NetBCE, a ten-layer interpretable deep neural network to predict linear BCEs. NetBCE substantially outperforms conventional ML methods, and reveals distinct features of BCEs [13].

Zhu et al. presented TripletGO, a novel hierarchical method to predict gene functions and specifically Gene Ontology (GO) terms by combining transcript expression profiles and protein homology inferences. TripletGO substantially improves the accuracy in predicting gene functions as compared to current state-of-the-art methods, in large part attributed to a novel triplet network method that can effectively boost function prediction using transcript expression profiles [14].

Last but not least, Wei et al. reported DrSim (similarity learning for drug discovery). As a learning-based framework, DrSim automatically infers similarity between transcriptional profiles. DrSim outperforms existing methods based on *in vitro* and *in vivo* datasets related to drug annotation and repositioning. DrSim may be useful for phenotypic drug discovery based on high-throughput transcriptional perturbation data [15].

Overall, the 15 articles in this special issue showcase the broad applications and powerful utilities of AI in omics. We anticipate that new breakthroughs in omics-driven biomedical research will be made by harnessing the enormous power of AI. GPB will continue to provide a platform for AI-based tools and discoveries in omics.

## CRediT author statement

**Feng Gao:** Writing - original draft, Writing - review & editing. **Kun Huang:** Writing - review & editing. **Yi Xing:** Writing - review & editing. All authors have read and approved the final manuscript.

## Competing interests

Yi Xing is a scientific cofounder of Panorama Medicine. Kun Huang has received research funding from Merck and Eli Lilly in the past three years. The authors declare no other competing interests.
